# Forecasting dynamic body weight of nonrestrained pigs from images using an RGB-D sensor camera

**DOI:** 10.1093/tas/txab006

**Published:** 2021-01-17

**Authors:** Haipeng Yu, Kiho Lee, Gota Morota

**Affiliations:** 1 Department of Animal and Poultry Sciences, Virginia Polytechnic Institute and State University, Blacksburg, VA, USA; 2 Division of Animal Sciences, University of Missouri, Columbia, MO, USA; 3 Center for Advanced Innovation in Agriculture, Virginia Polytechnic Institute and State University, Blacksburg, VA, USA

**Keywords:** body weight, computer vision, image analysis, swine

## Abstract

Average daily gain is an indicator of the growth rate, feed efficiency, and current health status of livestock species including pigs. Continuous monitoring of daily gain in pigs aids producers to optimize their growth performance while ensuring animal welfare and sustainability, such as reducing stress reactions and feed waste. Computer vision has been used to predict live body weight from video images without direct handling of the pig. In most studies, videos were taken while pigs were immobilized at a weighing station or feeding area to facilitate data collection. An alternative approach is to capture videos while pigs are allowed to move freely within their own housing environment, which can be easily applied to the production system as no special imaging station needs to be established. The objective of this study was to establish a computer vision system by collecting RGB-D videos to capture top-view red, green, and blue (RGB) and depth images of nonrestrained, growing pigs to predict their body weight over time. Over a period of 38 d, eight growers were video recorded for approximately 3 min/d, at the rate of six frames per second, and manually weighed using an electronic scale. An image-processing pipeline in Python using OpenCV was developed to process the images. Specifically, each pig within the RGB frame was segmented by a thresholding algorithm, and the contour of the pig was identified to extract its length and width. The height of a pig was estimated from the depth images captured by the infrared depth sensor. Quality control included removing pigs that were touching the fence and sitting, as well as those showing extremely distorted shape or motion blur owing to their frequent movement. Fitting all of the morphological image descriptors simultaneously in linear mixed models yielded prediction coefficients of determination of 0.72–0.98, 0.65–0.95, 0.51–0.94, and 0.49–0.93 for 1-, 2-, 3-, and 4-d ahead forecasting, respectively, of body weight in time series cross-validation. Based on the results, we conclude that our RGB-D sensor-based imaging system coupled with the Python image-processing pipeline could potentially provide an effective approach to predict the live body weight of nonrestrained pigs from images.

## INTRODUCTION

The average daily gain is important to enhance swine production as it can be used to determine animal growth rates and possible health challenges. Rapidly identifying changes in average daily gain is critical for efficient management of pig nutrition, feed efficiency, and detection of disease outbreaks. However, the labor-based measurement of live body weight using electronic scales is intensive, time-consuming, and may induce stress to pigs or cause injury to producers. Additionally, automatic scales typically integrated into feeding systems are still cost-prohibitive, and more than one animal may show up on the scale during weighing, which compromise the accuracy of the data. Reducing labor costs and enhancing welfare are expected to increase swine production and consequently secure the sustainability of meat production. Thus, developing new technologies that require less human involvement and are pig-friendly are essential.

Computer vision plays a pivotal role in accelerating phenotyping efforts by providing nonintrusive morphological measurements of animals with a temporal and spatial resolution (Morota et al., 2018). One example of computer vision systems is video-based high-throughput phenotyping by recording videos of animals as a sequence of images or frames (Van der Stuyft et al., 1991; Frost et al., 1997). The use of video-based high-throughput phenotyping technologies enables in collecting morphological traits more frequently with reduced workload and cost, thereby facilitating better pig management (Whittemore and Schofield, 2000; Tscharke and Banhazi, 2013). Earlier applications of computer vision systems to quantify pig morphological traits can be traced back to the late eighties to mid-nineties (Schofield, 1990; Minagawa and Ichikawa, 1994; Brandl and Jørgensen, 1996). These systems have been largely applied to monitor growth-related traits over time (Marchant et al., 1999; Schofield et al., 1999; Doeschl-Wilson et al., 2004, 2005; White et al., 2004; Banhazi et al., 2011). The advancement of imaging systems has led to the use of stereoscopy to estimate three-dimensional (3D) pig images (Minagawa, 1995; Wu et al., 2004; McFarlane et al., 2005; Shi et al., 2016), but the application of a stereo vision system requires multiple cameras with a detailed calibration process to synchronize them. Another 3D pig image reconstruction technology to estimate the live weight is the structure from motion; however, this has not yet been widely applied to swine (Pezzuolo et al., 2018b).

The recent availability of low-cost, off-the-shelf consumer 3D depth sensor cameras that provide additional depth information (third dimension) using infrared sensors, has greatly facilitated the development of 3D imaging system-based agricultural research (Rosell-Polo et al., 2015; Vázquez-Arellano et al., 2016). These depth sensor cameras apply structured light or time of flight technology to obtain depth data that can be used to estimate the height of a pig from a single top-view camera. This type of depth-sensing camera, also known as RGB-D camera if a red, green, and blue (RGB) sensor is jointly equipped, has been recently used to estimate the mass and size of pigs. For example, applications of these depth cameras include the ASUS Xtion series (Wang et al., 2018, 2019), Microsoft Kinect series (Kongsro 2014; Condotta et al., 2018, 2020; Pezzuolo et al., 2018a; Fernandes et al., 2019), and Intel RealSense series (Condotta et al., 2020).

Most studies deployed computer vision systems while restraining pigs at a weighing station or a feeder to facilitate data collection and subsequent image analysis. The approach can ensure that the pig is standing instead of lying or sitting, and that its posture is straight with as little bending or twisting as possible. However, given that pigs are typically housed in pens during pork production, it is more desirable to record videos while pigs are allowed to move freely within the pen. Although this creates a new challenge in terms of image analysis, videos collected from freely walking pigs will reflect a more practical scenario, because it is not required to immobilize the pigs each time they need to be weighed. Studies have reported body weight estimates from images of semirestrained (Wang et al., 2008a, 2008b) or nonrestrained (Kashiha et al., 2014; Jun et al., 2018) pigs automatically and from images of nonrestrained pigs manually (Wongsriworaphon et al., 2015; Buayai et al., 2019); however, depth images were not used in these studies. Furthermore, an RGB-D camera has not yet been used to collect time-series growth data by tracking the body weight measurements of the same pigs repeatedly and validate these data with statistical forecasting. Therefore, the objective of this study was to establish a computer vision system by collecting RGB-D videos and to develop an image-processing pipeline to automatically transform images of freely walking pigs into reliable, accurate, and biologically meaningful morphological image descriptors that can be used to predict live pig body weights.

## MATERIALS AND METHODS

All animal experiments were approved and carried out in accordance with the Virginia Tech Institutional Animal Care and Use Committee (IACUC) under protocol #19-182.

### Animals and Experimental Setup

A total of eight pigs housed in a swine facility at Virginia Tech were used to collect video image data over 38 d from early February to late March 2020. The pigs were a crossbreed of Yorkshire and Large White pigs. The grower entered the trial at 5 wk postweaning with the average (standard deviation) initial body weight of 23.4 (7.6) kg and their average final weight was 46.7 (8.7) kg at the end of the trial. A wide range of body weights was selected to test the robustness of the pipeline. The pigs were separated into four groups, each of which consisted of two pigs housed in a single pen (5 × 7 ft). The pigs were fed once a day. An imaging system was set up at the ceiling pipe of an indoor testing pen located in the middle of the facility. An Intel RealSense D435 camera (Intel, Santa Clara, CA, USA) was set perpendicularly to the floor and fixed at the ceiling pipe using a clamp. This depth-sensing camera was equipped with an RGB camera, an infrared camera, and an infrared emitter. Infrared coded structured light combined with stereo RGB matching technology was used to estimate the height of a pig. The distance between the camera and the floor was 2.25 m. The camera was controlled by a laptop computer via the Intel RealSense Viewer to capture RGB and depth images with a resolution of 848 × 480. A digital scale (Arlyn Scales, New York, NY, USA) was placed in a pen next to the image-recording pen to acquire pig body weight records manually. Both the scale-derived body weight records and the image data of each pig were collected daily in the afternoons.

### Data Collection

Manual body weight measurements. The digital scale was calibrated before obtaining the measurements. An assessor first recorded the pig identification data and then navigated the pig to the scale using a pig board. The scale-based body weight of each pig was recorded when the pig stood stably on the scale. The pig was then released to the adjacent image-recording pen to collect image data. All pigs were weighed repeatedly once per day during the growth period.

Image acquisition. A top-view video was obtained using the depth sensor camera while the pig was free to move within the pen. Each pig was recorded once per day for approximately 3 min/d, at a rate of six frames per second, totaling approximately 1,080 frames. The Intel RealSense Viewer was used to save all images and depth information as the Robot Operating System (ROS) bag files to optimize the storage. In total, 200 data points, including videos and corresponding scale-based body weight records of pigs, were collected. This resulted in more than 200,000 images to be processed.

Image processing. An automated image-processing pipeline was developed to acquire accurate and reproducible image descriptors. These image descriptors included length, width, and height derived from RGB and depth images. A unique challenge in image processing was that pig movement resulted in nonstationary images. OpenCV, a library in Python (Bradski, 2000), coupled with data science libraries NumPy (Virtanen et al., 2020) and Pandas (Reback et al., 2020), was used for image analysis. The details of the image-processing pipeline are described below.

**Figure 1.**
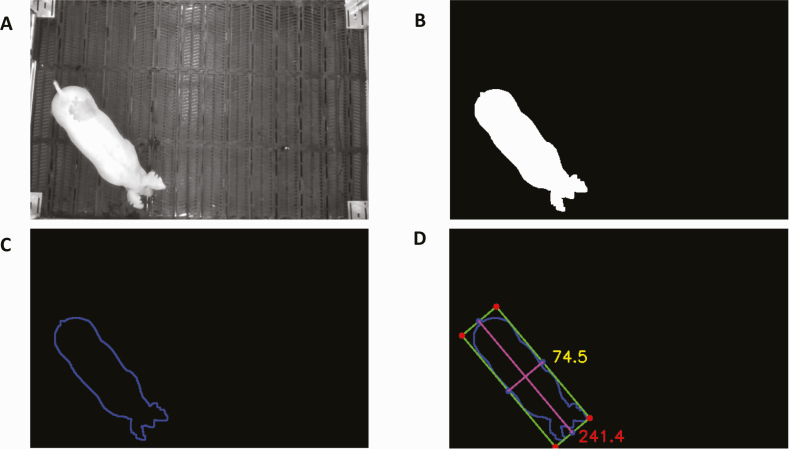
A flow of extracting pig length and width from RGB images. (A) Grayscale image converted from RGB image. (B) Segmented image coupled with morphological opening. (C) Contour of the segmented image. (D) Bounding box of the contour image.

Extracting length and width from RGB images. The ROS bag video files were converted to RGB images in a portable network graphics (PNG) format using the Intel RealSense software development kit 2.0. For each frame, the RGB image was converted to a single-channel grayscale image (Figure 1A). The region occupied by the pig was segmented from the grayscale image. In this step, we applied thresholding to separate the pig from the background by identifying the largest connected object in the image. This was followed by performing a morphological opening to remove the tail from the segmented pig image (Figure 1B). We then extracted the contour of the segmented pig (Figure 1C). Here, the contour referred to a line that described the shape of the segmented pig and consisted of edge points. Contour coordinates from the contour image were then obtained to draw a rotated bounding box of the pig (Figure 1D). A quality control step included checking whether the pig was attached to the border. A frame was removed from the analysis if its rotated bounding box was attached to the fence. If the pig was not attached to the border, the length and width of the rotated bounding box were calculated in terms of pixels to represent the spine length (Kollis et al., 2007) and width of the detected pig. If the pixel count of the pig region was lower or higher than the predefined threshold, the corresponding frames were discarded because they were likely to include severe shape distortion or motion blur owing to pig movement. All RGB frames were processed according to these steps. Instead of selecting the single best frame, the width and length estimate medians were calculated from all frames to derive the single final estimates for each video. The median was chosen so that the final estimates were robust against outliers owing to shape distortion or motion blur resulting from pig movement.

**Figure 2.**
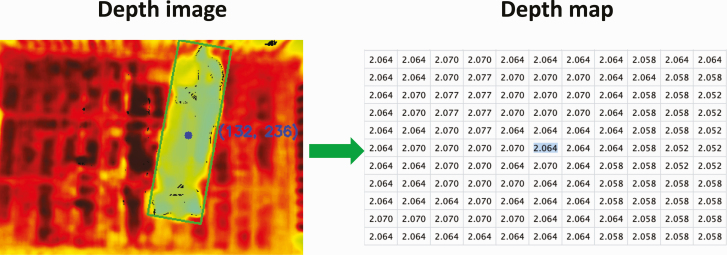
A flow of extracting pig height from depth images. The depth image was used to identify the centroid coordinate of a pig. This coordinate was used to obtain the distance or depth map value between the pig surface to the camera using the depth map. Similarly, the distance between the ground to the camera was obtained using the depth image and depth map. The height of the pig was estimated by subtracting these two distances.

Acquiring pig height from depth images. The depth sensor camera employed infrared coded structured light technology, in which each pixel value was associated with the distance from the camera. For each pig, the ROS bag video file was converted into a PNG format depth image and the corresponding depth map that included numerical distances between the sensor and the pig or between the sensor and the background for each image pixel in metric units. Each infrared-derived depth image was first converted to a single-channel hue image using the hue, saturation, and value (HSV) transformation. After some experiments, this color space yielded the best result by correctly separating pigs from the background. Then, the pig was segmented following the same procedure as described for the RGB images (Figure 1B). The centroid coordinate of the segmented pig in the depth image was identified to obtain the distance of the camera from the pig in the depth map (Figure 2). By subtracting the distance from the camera to the ground (i.e., 2.25 m) and the distance from the camera to the centroid of the segmented depth image, we obtained a pig height estimate from the top-view camera. All the depth frames were processed according to these steps. However, as pigs were allowed to move freely during video recordings, their positions could have varied over time. In order to distinguish whether a pig was standing or sitting, *k*-means clustering was performed on the height estimates by setting the number of clusters equals to two. The cluster with the highest median value was considered as the estimate of height when the pigs were standing. The median of this cluster was calculated to derive the single final height estimate for each video.

### Statistical Analysis

**Figure 3.**
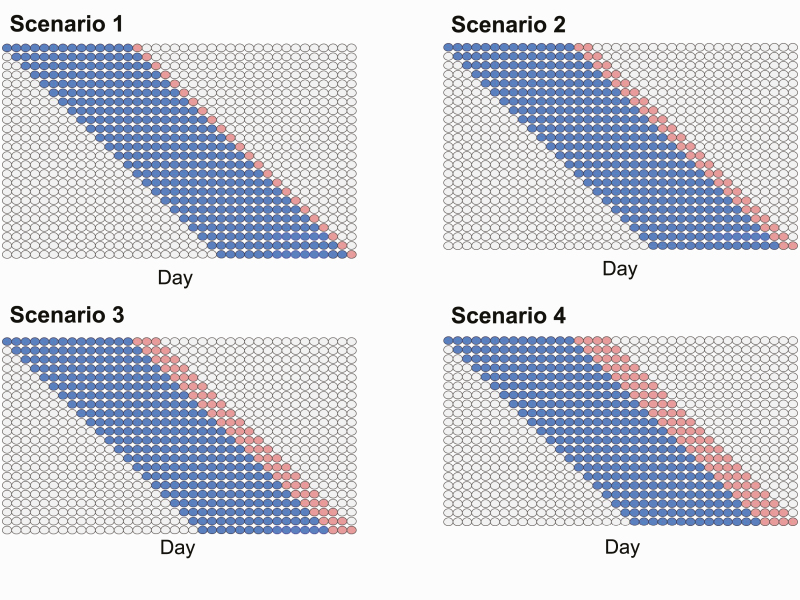
Time-series cross-validation scenarios. Scenario 1: One-day ahead forecasting from the most recent 14 d. Scenario 2: Two-day ahead forecasting from the most recent 14 d. Scenario 3: Three-day ahead forecasting from the most recent 14 d. Scenario 4: Four-day ahead forecasting from the most recent 14 d. Blue and red points represent the training and testing sets, respectively.

The relationship between manual body weight measurements and morphological image descriptors (length, width, and height) was analyzed statistically. A volume image descriptor was further derived by multiplying the length, width, and height. We first estimated pairwise Pearson’s correlations between manual body weight measures and the four image descriptors. As each pig was weighed and video recorded repeatedly during the experiment, the observations constituted growth data. Thus, the predictive performance of the image descriptors was evaluated using a move forward time-series cross-validation scheme, as is shown in Figure 3. The most recent 14 d were trained to forecast 1-, 2-, 3-, and 4-d ahead body weights by sliding the training data by one. We quantified the prediction performance of length, width, and height morphological image descriptors using a linear mixed model (Bates et al., 2015). The image descriptor of the volume was not considered, in order to avoid multicollinearity. A random intercept model including fixed morphological image descriptors and the random animal effect was used. Forecasting was performed using either the image descriptors alone (LMM1) or both the image descriptors and the animal effect (LMM2). The prediction performance was evaluated by the prediction coefficient of determination (*R*^2^). The R package lme4 was used for statistical analysis (Bates et al., 2015).

## RESULTS

### Relationships Between Body Weight Records and Image Descriptors

**Figure 4.**
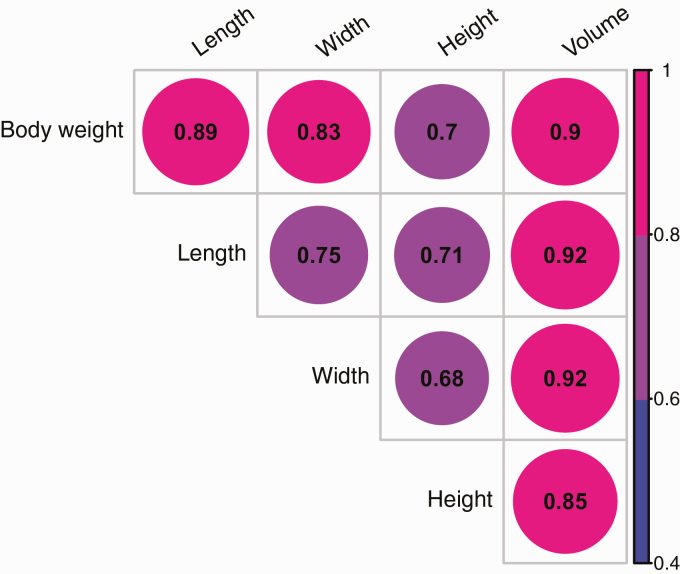
Heat map of Pearson’s correlation coefficients between manually measured body weight records from the digital scale and morphological image descriptors.

The pairwise Pearson’s correlation heat map between manually measured body weight and each image descriptor across all time points is reported in Figure 4. The image descriptor volume had the highest correlation with body weight (0.90), followed by length (0.89), width (0.83), and height (0.70). Among all the image descriptors, the highest correlation was obtained between length and volume and width and volume (0.92), whereas width and height had the lowest correlation coefficient (0.68).

### Prediction Performance

**Figure 5.**
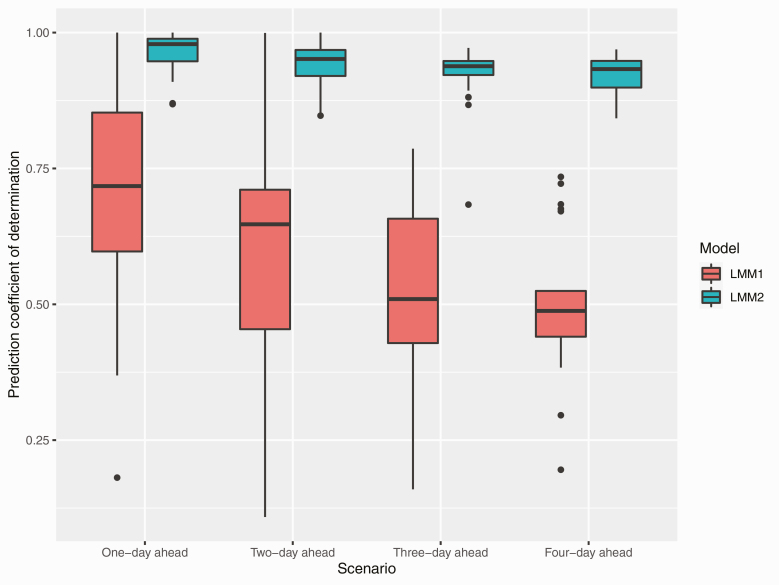
Prediction coefficient of determination from linear mixed models (LMM) forecasting 1-, 2-, 3-, and 4-d ahead body weight from the most recent 14 d. The random intercept model was fit for LMM1 and LMM2 including the three fixed morphological image descriptors and random animal effect. Forecasting was made using either the image descriptors alone (LMM1) or the image descriptors and animal effect (LMM2).

The prediction performance of the image descriptors is presented in Figure 5. Time series cross-validation based on a window size of 14 d was used to derive the prediction *R*^2^. Fitting all of the morphological image descriptors simultaneously in linear mixed models yielded *R*^2^ of 0.72–0.98, 0.65–0.95, 0.51–0.94, and 0.49–0.93 for 1-, 2-, 3-, and 4-d ahead forecasting, respectively, of body weight. The best prediction was achieved when the random intercept model coupled with the image descriptors and random animal effect was used for forecasting (LMM2). The use of three image descriptors alone for forecasting yielded lower prediction performance and less stable results according to cross-validation uncertainty (LMM1). Overall, 1-d ahead forecasting resulted in the highest prediction performance. The prediction performance decreased as the forecasted day was further away from the training set. Four-day ahead forecasting resulted in the lowest prediction performance.

## DISCUSSION

The size and shape of animals measured over time are of great importance as they can reflect the animal growth rate, feed efficiency, and health status. Live body weight is typically measured manually or through electronic scales; however, the approach can be labor intensive and may cause stress to animals. Therefore, the use of imaging systems offers an alternative approach that does not require direct contact with the animal. We used an RGB-D camera to build an advanced computer vision system to capture morphological measures to predict pig body weight from RGB and depth video images. However, an important challenge is that image data are not yet phenotypes. It is necessary to process and extract meaningful traits from images. A Python-based image-processing pipeline was developed in this study to obtain morphological image descriptors automatically.

There are a few factors that affect the accuracy of RGB and depth images. The most notable factor in this study was that pigs were not static or immobilized but allowed to move freely in a pen. Nonstationary imaging has brought a new challenge in image processing. For example, a two-thirds reduction in height repeatability has been reported in walking dairy cows compared to still-standing cows, during image processing (Salau et al., 2017). As the ideal pig posture was hardly obtained in the current study, extra steps were added as a quality control process to remove pig images that were touching the fence and sitting as well as those showing extremely distorted shape, or motion blur due to their movement. These filtering steps decreased the number of low-quality images arising from the dynamic movement of pigs, but somehow still influenced the image segmentation results.

The second factor was the distance between the sensor and the pig. It has been reported that random error measurements increase with increasing distance between the sensor and the object (Condotta et al., 2020). In this study, the distance between the camera and the floor was 2.25 m, which made it possible to capture the entire pen. Previous studies have reported shorter distances than that utilized in the present study, for example, 1.7 m above the ground (Condotta et al., 2020) or 1.0–1.2 m above the pig (Pezzuolo et al., 2018a). A longer camera clamp may be required to reduce the distance to be either equal to or less than 2 m in future experiments to obtain more accurate depth data. Although the distance between the camera and the ground is not always reported in livestock computer vision studies, this information should be shared more frequently for comparison purposes. In this study, the light intensity or illumination variation was of less concern, as the sensor camera was set up in an indoor housing system.

The third factor is the image processing of RGB data. In the majority of previous studies that utilized RGB-D cameras, only depth images were analyzed to extract morphological image descriptors such as length, width, height, or volume (Kongsro 2014; Condotta et al., 2018, 2020; Fernandes et al., 2019). The use of depth images alone was not a viable option in the current study because their quality was not sufficient to estimate length and width, partly owing to motion blurs and the relatively small size of pigs (early growers). This was why RGB images were processed to obtain length and width. Depth images were used to estimate height, while volume was estimated from a combination of RGB and depth images in this study.

The highest *R*^2^ value of 0.98 obtained from 1-d forecasting (LMM2) was similar to those obtained from recent studies using RGB-D cameras on restrained pigs (e.g., 0.94 (Fernandes et al., 2019), 0.95 (Pezzuolo et al., 2018a), and 0.99 (Condotta et al., 2018)). This value corresponded to 2.2 kg of mean absolute error. In this case, however, we fitted both the image descriptors and the animal effect in the training set used for forecasting. When both the image descriptors and the animal effect were trained, but only the image descriptors were used for prediction, the *R*^2^ value decreased to 0.72 (LMM1). This result was similar to the reported *R*^2^ value of 0.79 when pigs were likewise not restrained (Jun et al., 2018). The predicted power in the longitudinal data is a function of morphological image features and individual animal growth patterns. Accounting for individual animal variation across time via random animal effects in the testing set was critical to improve forecasting results. An intermediate body weight forecast may allow us to perform interventions if required. However, caution should be exercised because, in the aforementioned studies, each pig was measured only once, and time-series cross-validation was not employed. Collectively, the choice of depth cameras and statistical methods, and the aforementioned three factors contributed to the differences observed between our results and those of recent studies.

To the best of our knowledge, the present study is the first to predict live body weight of nonrestrained pigs over time using an RGB-D camera. Depth sensor technology is advancing rapidly, thus sensor cameras quickly fade from state-of-the-art to out-of-date. For example, Kinect cameras versions 1 and 2, and Xtion Pro, which have been widely used in recent studies, are no longer available on the market (Kongsro 2014; Condotta et al., 2018; Jun et al., 2018; Fernandes et al., 2019). On the other hand, the RealSense camera used in the current study can be purchased in 2020. We believe that the image acquisition and image-processing steps reported here could be useful for anyone interested in using RealSense depth sensor cameras for performing image-based livestock phenotyping.

Our results showed that we could predict the daily weight gain of pigs nonintrusively from RGB and depth image data, while preventing frequent contact between animal take care person and pigs. However, three challenges should be taken into account in future research. The first challenge is extending the scope of this research to include multiple pigs in a single image. The analysis of multiple pigs requires animal identification and tracking. The second challenge is to automate the image acquisition process. Although the image-processing pipeline was automated in the present study, the image acquisition process involved a manual step for controlling the video recordings. The third challenge is that body weight itself may not reflect, to great extent, the actual fat and lean content of pigs, both of which are critical parameters in swine production (Whittemore and Schofield, 2000). In such a case, not only a top-view camera, but also a side-view depth camera may be required to reconstruct the 3D surface area of pigs, in order to obtain more quantitative body conformation data. Image-based phenotyping can be classified as one of the recently emerging precision livestock farming technologies (Vranken and Berckmans, 2017; Benjamin and Yik, 2019). We contend that RGB-D camera-based phenotyping can be used to collect body weight data more frequently than before and enhance swine production efficiency by promoting precision livestock farming.
